# ZDHXB-101 (3′,5-Diallyl-2, 4′-dihydroxy-[1,1′-biphen-yl]-3,5′-dicarbaldehyde) protects against airway remodeling and hyperresponsiveness via inhibiting both the activation of the mitogen-activated protein kinase and the signal transducer and activator of transcription-3 signaling pathways

**DOI:** 10.1186/s12931-020-1281-x

**Published:** 2020-01-13

**Authors:** Jun-xia Jiang, Hui-juan Shen, Yan Guan, Yong-liang Jia, Jian Shen, Qi Liu, Qiang-min Xie, Xiao-feng Yan

**Affiliations:** 1 The Second Affiliated Hospital, Zhejiang University School of Medicine, # 88 Jiefang Rd, Hangzhou, 310009 Zhejiang Province China; 2 Zhejiang Respiratory Drugs Research Laboratory of State Food and Drug Administration of China, Zhejiang University School of Medicine, # 866 Yuhangtang Rd, Hangzhou, 310058 Zhejiang Province China; 3 Affiliated Sir Run Run Shaw Hospital, Zhejiang University School of Medicine, Hangzhou, China

**Keywords:** Epoxyeicosatrienoic acid, Soluble epoxide hydrolase, Asthma, Airway remodeling, Airway hyperresponsiveness

## Abstract

Airway remodeling consists of the structural changes of airway walls, which is often considered the result of longstanding airway inflammation, but it may be present to an equivalent degree in the airways of children with asthma, raising the need for early and specific therapeutic interventions. The arachidonic acid cytochrome P-450 (CYP) pathway has thus far received relatively little attention in its relation to asthma. In this study, we studied the inhibition of soluble epoxide hydrolase (sEH) on airway remodeling and hyperresponsiveness (AHR) in a chronic asthmatic model which long-term exposure to antigen over a period of 12 weeks. The expression of sEH and CYP2J2, the level of 14, 15-epoxyeicosatrienoic acids (EETs), airway remodeling, hyperresponsiveness and inflammation were analyzed to determine the inhibition of sEH. The intragastric administration of 3 or 10 mg/kg ZDHXB-101, which is a structural derivative of natural product honokiol and a novel soluble epoxide hydrolase (sEH) inhibitor, daily for 9 weeks significantly increased the level of 14, 15-EETs by inhibiting the expression of sEH and increasing the expression of CYP2J2 in lung tissues. ZDHXB-101 reduced the expression of remodeling-related markers such as interleukin (IL)-13, IL-17, MMP-9 N-cadherin, α-smooth muscle actin, S100A4, Twist, goblet cell metaplasia, and collagen deposition in the lung tissue or in bronchoalveolar lavage fluid. Moreover, ZDHXB-101 alleviated AHR, which is an indicator that is used to evaluate the airway remodeling function. The inhibitory effects of ZDHXB-101 were demonstrated to be related to its direct inhibition of the extracellular signal-regulated kinase (Erk1/2) phosphorylation, as well as inhibition of c-Jun N-terminal kinases (JNK) and the signal transducer and activator of transcription-3 (STAT3) signal transduction. These findings first revealed the anti-remodeling potential of ZDHXB-101 lead in chronic airway disease.

## Highlights

- ▶ ZDHXB-101 is a novel soluble epoxide hydrolase (sEH) inhibtor.
- ▶ ZDHXB-101 increases the CYP2J2 expression and 14, 15-EET level.
- ▶ ZDHXB-101 relives the airway remodeling and airway hyperresponsiveness.
- ▶ Effect of ZDHXB-101 is related to down-modulate MAPK and STAT3 pathways.

## Background

Asthma is characterized by reversible airflow obstruction [1, 2]. Airway remodeling is one crucial part of the reversible airflow obstruction of asthma [3], including goblet cell metaplasia (GCM), epithelial-to-mesenchymal transition (EMT), excessive subepithelial collagen deposition, airway smooth muscle hyperplasia, and increased vascularity [4, 5]. Although the prevailing thought is that remodeling is an abnormal response to persistent airway inflammation, recent evidence, especially from studies of remodeling in asthmatic children, suggests that the two processes occur in parallel [6, 7]. The effects of asthma therapy on airway remodeling are not been studied extensively due to the challenges of obtaining airway tissue in the context of clinical trials. Corticosteroids remain the cornerstone of asthma therapy because of their effects on inflammatory and structural cells, and their effects on remodeling have been better studied than those of other drugs [8, 9]. Bronchial thermoplasty is the only asthma therapy to primarily target remodeling, although how it results in the apparent clinical benefits seen is not exactly clear [10]. Early interventions to prevent remodeling may possibly help prevent the development of asthma but much remains to be studied about this possibility. However, we still do not know how to efficiently prevent airway remodeling. Therefore, it is urgent to explore these mechanisms and find reliable drugs for the prevention and treatment of the airway remodeling processes underlying asthma pathology [8–10].

Cytochrome P450 2 J2 (CYP2J2) is a known arachidonic acid (AA) epoxygenase that mediates the formation of four bioactive regioisomers of *cis*-epoxyeicosatrienoic acids (EETs). CYP2J2 expression is primarily expressed in the small intestine, pancreas, lung, and heart. Changes in CYP2J2 levels and activity in disease states or polymorphisms are proposed to lead to dysfunction in various organs. The major CYP2J2 products are 14,15-EETs. It has been previously shown that 14,15-EETs have important biological effects including anti-inflammation and anti-remodeling. Therefore, in our study we chose to study the anti-inflammation and anti-remodeling effects of 14,15-EETs [11, 12]. All EETs, including 14,15-EETs, are metabolized to the less active dihydroxyeicosatrienoic acids by soluble epoxide hydrolase (sEH) [13]. Earlier studies have demonstrated that chronic treatment of spontaneously hypertensive rats with an sEH inhibitor, or mice with deletion of the gene responsible for the production of the sEH enzyme, improved vascular remodeling [14]. Recently, Hammock’s lab reported that the administration of an sEH inhibitor attenuates allergic airway inflammation and airway responsiveness in a murine model [15]. Therefore, we hypothesized that sEH inhibitors could be used in the treatment of airway remodeling. In our preliminary study, ZDHXB-101 (chemical structure in Additional file 1: Figure S1), a honokiol derivative, was found to have a significant inhibitory effect on airway hyperresponsiveness in the mouse model of asthma, but the target was unclear. Further study revealed that this compound is a strong inhibitor of sEH. Further study revealed that this compound is a strong inhibitor of sEH. In our preliminary result for human recombinant sEH, 50% inhibitory rate (IC_50_) of ZDHXB-101 was 1.23 nM. ZDHXB-101 (5, 10, and 20 μM) suppressed cell proliferation in 16HBE cells in a concentration-dependent manner (Additional file 1: Figure S2A and B), but it did not inhibited cell activity (Additional file 1: Figure S2C). ZDHXB-101 (10 μM) reversed the TGF-β1-induced an increase in sEH protein expression in 16HBE cells (Additional file 1: Figure S2D and E). The treatment of cells with ZDHXB-101 not only resulted in significantly increased EETs levels, but also reversed effect of TGF-β1 compared with that of the vehicle group (Additional file 1: Figure S2F). Based on these results, we hypothesized that ZDHXB-101 may be involved in the airway remodeling and hyperresponsiveness (AHR) of asthma by modulating the endogenous EETs levels. However, there is no report on whether ZDHXB-101 can be used to inhibit airway remodeling, AHR or inflammation, and the possible mechanisms remain yet to be established. Molecular studies suggest that the mitogen-activated protein kinases (MAPK) family [16, 17] and signal transducer and activator of transcription (STAT) 3, in addition to other pathways, play pivotal role in regulating allergic airway remodeling and inflammation in asthma under various contexts [18].

In the present study, we evaluated the effects and the possible mechanisms of ZDHXB-101 on airway remodeling in a chronic asthmatic model. We found that ZDHXB-101 inhibited airway remodeling and AHR by inhibiting sEH, and activating the expression of CYP2J2, and decreasing the phosphorylation of the Erk1/2, JNK and STAT3 signaling pathway proteins. These results demonstrated that ZDHXB-101 may be a potential therapeutic agent for asthma.

## Materials and methods

### Materials

ZDHXB-101 (3′,5-Diallyl-2, 4′-dihydroxy-[1,1′-biphen-yl]-3,5′-dicarbaldehyde); (C_20_H_20_O_4_; MW: 324.35; purity> 98%), which is a structural analogues of the natural product honokiol, was kindly provided by Prof. Yuqing Zhao from Shenyang Pharmaceutical University. The structure of ZDHXB-101 is shown in Additional file 1: Figure S1. Ovalbumin (OVA) was purchased from Sigma-Aldrich (St. Louis, MO). Primary antibodies against GAPDH (Santa Cruz Biotechnology, Santa Cruz, CA); sEH (Abcam, Cambridge, MA, USA); p-Erk1/2, Erk1/2, p-JNK, JNK, p-p38, p38, p-STAT3, and STAT3 (Cell Signaling Technology, Danvers, MA) were used in the immunoblotting analysis. Mouse IL-13, IL-17 and MMP-9, and 14, 15-EET/DHET enzyme-linked immunosorbent assay (ELISA) kits were purchased from eBioscience (San Diego, CA), and R&D (Detroit, MI, USA). All of the other reagents and preparations were obtained as indicated.

### Animals

All of the animal experiments were performed strictly in accordance with international ethical guidelines and the National Institutes of Health Guide concerning the Care and Use of Laboratory Animals. Female ICR mice (weighing 20 ± 2 g) were purchased from Shanghai Slac Laboratory Animal Co. Ltd. (NO. SCXK 2012–0002). The mice were housed for 7 days so that they could adapt themselves to the environment before the experiments. The animals were housed in isolated ventilated cages (4–5 mice/cage) and maintained at 20–23 °C under a 12 h light/12 h dark cycle and 45–65% humidity. Standard laboratory food and water were provided ad libitum. All of the animal procedures and experiments that were conducted in this study were approved by the Ethics Committee of the Zhejiang University, School of Medicine (Permit No. ZJU20160274). All of the animals received humane care in accordance with the guide prepared by the Committee of the Care and Use of Laboratory Animals of Zhejiang University.

Forty ICR mice were randomly divided into four groups, with 10 mice in each group, including the control group, OVA model group, OVA + ZDHXB-101 (3 mg/kg) group, and OVA + ZDHXB-101 (10 mg/kg) group.

### Chronic asthmatic model and therapeutic intervention

**Fig. 1:**
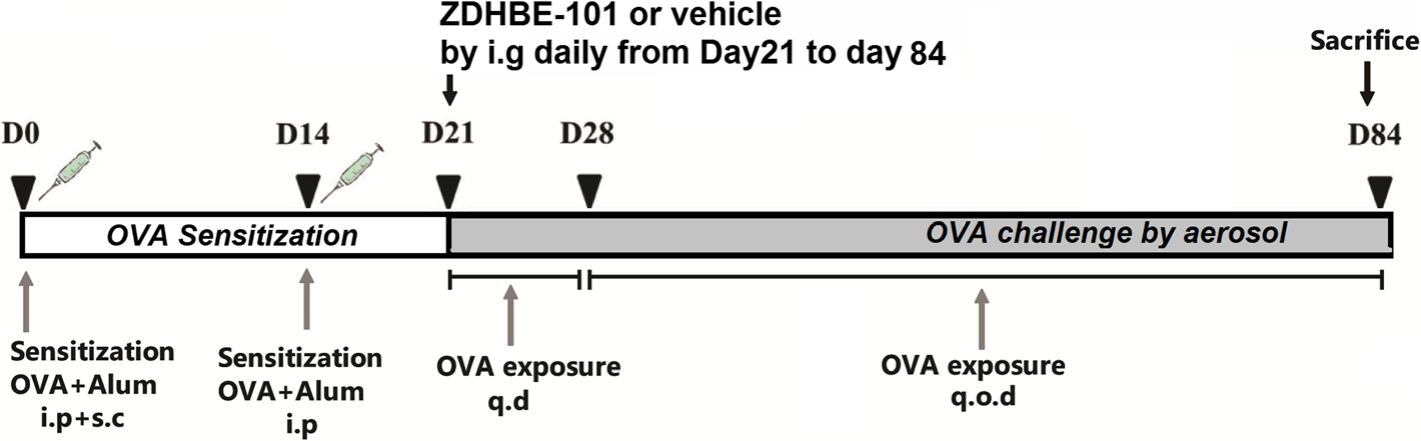
The ovalbumin sensitization, challenge, and drug treatment procedures. ICR mice were sensitized with ovalbumin (OVA) and aluminum hydroxide gel by intraperitonealy (i.p) and subcutaneously (s.c) injections at days 0 and 14, and an OVA (10 mg/ml) challenge was performed by aerosol for 30 min per day (q.d) from day 21 to day 28. The mice continued to be exposed to inhaled OVA (10 mg/ml) for 30 min every other day (q.o.d) from day 29 to day 84. The control mice were sensitized and challenged only with saline. ZDHXB-101 or a vehicle (CMC-Na) was given by intragastric administration (i.g) 30 min before each OVA challenge from day 21 to day 84

To sensitize the mice, 2 mg OVA (Grade V, Sigma-Aldrich Chemical Co., St. Louis, MO) were dissolved in 10 mg aluminium hydroxide gel adjuvant in 1.0 ml saline. Each mouse was administered an intraperitoneal (i.p) with 0.5 ml, and subcutaneously (s.c) injected with 0.05 ml/site at four footpads (4 sites), the neck (one site), back (3 sites), and two groins (2 sites) on experimental day 0. Normal control mice were injected with only the aluminium hydroxide gel adjuvant in saline following the same protocol. On day 14, all of the mice except those in the control group were sensitized again with an intraperitoneal injection of 2 mg OVA mixed with 1% aluminium hydroxide gel solution. Then, from day 21, the mice were challenged by an aerosolized formulation of 10 mg/mL OVA in normal saline for 30 min, and this process was repeated daily until day 28 after sensitization. From day 28, the mice were challenged 3 days per week for another 8 weeks. In the control group, saline replaced OVA during the sensitization and challenge. Half an hour before each challenge, all of the mice except those in the control and OVA groups were intragastrically administered with 3 or 10 mg/kg ZDHXB-101, and the mice in control and OVA groups were intragastrically administered the same volume of with vehicle (0.5% CMC-Na) in the same volume. The OVA sensitization, challenge, and drug treatment procedure is shown in Fig. 1.

### Determination of airway hyperresponsiveness (AHR)

A commercially available plethysmograph, acquisition software, and mouse-sized plethymograph chambers (Buxco Electronics, Troy, NY), were used for the total pulmonary airflow analysis in unrestrained conscious mice. This system allows for the measurement of the differential pressure within the chambers that is caused by the animal breathing. Pressure differences between the chambers containing individual animals and a reference chamber were used to extrapolate minute volume, tidal volume, breathing frequency, and enhanced pause (Penh) [19, 20]. Penh is a function of the total pulmonary airflow during the respiratory cycle and is described by the following equation: Penh = [(Te/Tr-1) (PEF/PIF)], where PEF is the peak expiratory pressure, PIF is the peak inspiratory pressure, and pause is a component of the expiration time (Te/Tr-1). This parameter is dependent on the breathing pattern and correlates with airway resistance, which is measured by traditional invasive techniques with ventilated mice [19, 20]. Pulmonary function was assessed by whole-body plethysmography after the last OVA inhalation, as according to the previous studies [15]. Mice were placed in measured chambers for 10 min prior to the evaluations. Aerosolized normal PBS or increasing concentrations (3.125–50 mg/ml) of methacholine (Mch) were nebulized via a chamber inlet for 90 s. Recording and baseline measurement were averaged for 3 min after each nebulization of varying methacholine concentration. Airway resistance was expressed as Penh.

### The preparation of bronchoalveolar lavage fluid (BALF) and cell counting

BALF were obtained by cannulating the trachea and flushing the lung three times with 0.5 ml of PBS containing 1% BSA and 5000 IU/l heparin, and the total cell number was determined. The BALF was concentrated by centrifugation once with PBS containing 2% FCS (1000 rpm, 10 min at 4 °C), after which the supernatant was stored at − 80 °C and was analyzed by ELISA. The cell pellet was resuspended in PBS, and the total cell count in the BALF was determined with a Neubauer chamber. To perform the differential leukocyte cell count, 10 μl of the cell suspension were transferred to a glass slide and stained with Wright-Giemsa stain. Two hundred cells from the BALF were counted under a light microscope, and the cell differentials were determined by counting the number of eosinophils, macrophages and lymphocytes. The total number of each cell type was determined by multiplying the percentage of each cell type by the total number of cells.

### Histopathological and immunohistochemical examination

The bottom of the left lung tissue samples were fixed in 10% formalin and were then dehydrated by washing with ascending grades of ethanol. Then, the samples were embedded in paraffin wax and sectioned into 3–4 μm. The lung sections were prepared for routine hematoxylin-eosin (H&E), periodic acid–schiff (PAS) and Masson’s trichrome stains. An H&E stain was used to evaluate inflammatory cell infiltration using light microscopy based on the peribronchial cell counts and the severity of the infiltration of inflammatory cells. A 5-point scoring system was used as described previously [21]. Goblet cell hyperplasia was assessed by staining with PAS. To determine the extent of mucus production, the percentage of PAS -positive staining cells in the airway epithelium was quantified [21]. Collagen deposition around the bronchial airway was observed by Masson’s trichrome staining. The severity of collagen deposition was evaluated by the Image pro 6.1 software system and was determined from the positive staining area. The criterion for grading the severity of airway remodeling, includes the thickness of the airway smooth muscles, collagen deposition; and an immunohistochemistry analysis of airway remodeling markers including N-cadherin, α-SMA, S100A4 and MMP-9. Under 200× magnification, each section was randomly selected and photographed for 5 fields. An immunohistochemistry analysis was carried out according to the protocol provided with the Streptavidin-Biotin Complex kit (Boster Bio-engineering Ltd. Co., Wuhan, China), The primary antibodies against sEH, N-cadherin, α-SMA, MMP-9and S100A4 (1:200 in dilution) were incubated overnight at 4 °C. The immunocomplexes were visualized with 3,3′-diaminobenzidine (DAB) and measured with the DP2-BSW software (Olympus, Tokyo, Japan). The staining was quantified using Image Pro 6.1 software. All of the analyses were performed blinded.

### Measurement of 14, 15-EETs, IL-13, IL-17 and MMP-9 by ELISA

To investigate the effects of ZDHXB-101 on the levels of 14, 15-EETs in the mouse lungs of the OVA-induced allergic model, lung tissues were collected 24 h after the last challenge. The level of 14, 15-EETs in the supernatant of homogenized lung tissue homogenization was measured by ELISA according to the manual. To investigate the effects of ZDHXB-101 on inflammatory cytokines and matrix metalloproteinase levels in the mouse lungs of the OVA-induced allergic model, BALF were collected 24 h after the last challenge. The concentrations of IL-13, IL-17 and MMP-9 in the BALF were detected and analyzed with the ELISA kits using paired matched antibodies according to the manufacturer’s instructions. The color absorbance of the samples at 450 nm was determined using a Bio-Rad microplate reader. The concentrations of 14, 15-EETs, IL-13, IL-17 and MMP-9 were calculated by generating a standard curve using standard proteins and were analyzed using curve expert 1.3 software.

### RNA isolation and quantitative PCR (qRT-PCR)

Total RNA was extracted from lung homogenates using TRIzol Reagent (Takara, TaKaRa Biotechnology, Dalian, China) according to the manufacturer’s instructions. First-strand cDNA was generated from 4 μg of total RNA using oligo-dT to prime the reverse transcription reaction according to the manufacturer’s protocol. The PCR primers were purchased from Shanghai Bioengineering Ltd. (Shanghai, China). All of the primers were checked against the basic local alignment search tool for selectivity. Real-time PCR cycling was performed using a Real-Time PCR System 7500 (Applied Biosystems, Carlsbad, CA, USA). The PCR reaction mixture consisted of 10.4 μL of 2× SYBR Green 1 Master Mix, 0.4 μL of both sense and antisense primers, 2.0 μL of sample cDNA solution, and double distilled water to obtain a final volume of 20 μL. The program was conducted as follows: a denaturation step at 95 °C for 10 min, followed by 40 cycles of 95 °C for 15 s, then 60 °C for 1 min. β-actin was amplified as an internal control. The mRNA levels were calculated using the 2^-ΔΔCt^ method (relative) [22]. The primer sequences are shown in Table 1.

**Table 1:** Primer sequences used in the present study

Genes	Primer Sequences (5′-3′)
IL-13	Sense: CCTCTGACCCTTAAGGAGCTTAT
	Antisense: CGTTGCACAGGGGAGTCT
IL-17	Sense: GAGAAGATGCTGGTGGGTGT
	Antisense: TTTCATTGTGGAGGGCAGAC
MMP-9	Sense: GTATGGTCGTGGCTCTAAGC
	Antisense: AAAACCCTCTTGGTCTGCGG
N-Cadherin	Sense: CGGTGCCATCATTGCCATCCT
	Antisense: AGTCATAGTCCTGGTCTTCTTCTCCT
Twist	Sense: TACGCCTTCTCCGTCTGG
	Antisense: CTAGTGGGACGCGGAC
β-actin	Sense: GGCTGTATTCCCCTCCATC
	Antisense: ATGCCATGTTCAATGGGGTA

### Western blot analysis

The tissue from the different groups were lysed in a radioimmunoprecipitation assay (RIPA) buffer (Biyuntian Biotechnology, Haimen, China) containing 1% PMSF (Haoxin Biotechnolgy, Hangzhou, China) and 1% protease inhibitors and were then denatured. The total protein concentration was determined by the BCA Protein Assay Kit (CWbiotech, Beijing, China). Equal quantities (30 μg) of proteins samples were separated by SDS- polyacrylamide gels. The proteins were then transferred to 0.45 μm nitrocellulose membranes. The membranes were blocked with 5% fat-free milk, and incubated with the CYP2J2 polyclonal antibody (1:1000, Invitrogen, USA), p-Erk1/2, Erk1/2, p-JNK, JNK, p-p38, p38, p-STAT3, STAT3 antibodies (1:1000, Cell Signaling Technology, Beverly, MA, USA) or GAPDH antibody (1:1000, Santa Cruz Biotechnology, Santa Cruz, CA) respectively, at 4 °C overnight. After, the blots were incubated with goat anti-rabbit 800 antibodies (1:5000) for 2 h at room temperature. The immunoreactive bands were visualized by a two-color infrared imaging system (Odyssey; LI-COR, Lincoln, NE, USA).

### Statistical analysis

GraphPad prism V5.0 software (Graphpad prism, CA, USA) was used for the statistical analyses. The data are presented as the means ± S.E.M. The statistical tests were performed using SPSS software (version 16.0; SPSS Inc., Chicago, IL, USA). The differences between the mean values of multiple groups were analyzed by a one-way analysis of variance (ANOVA), followed by the Student-Newman-Keuls test. Statistical significance was set at *P* < 0.05.

## Results

### ZDHXB-101 suppresses sEH expression and increases CYP2J2 expression and 14,15-EET levels

**Fig. 2:**
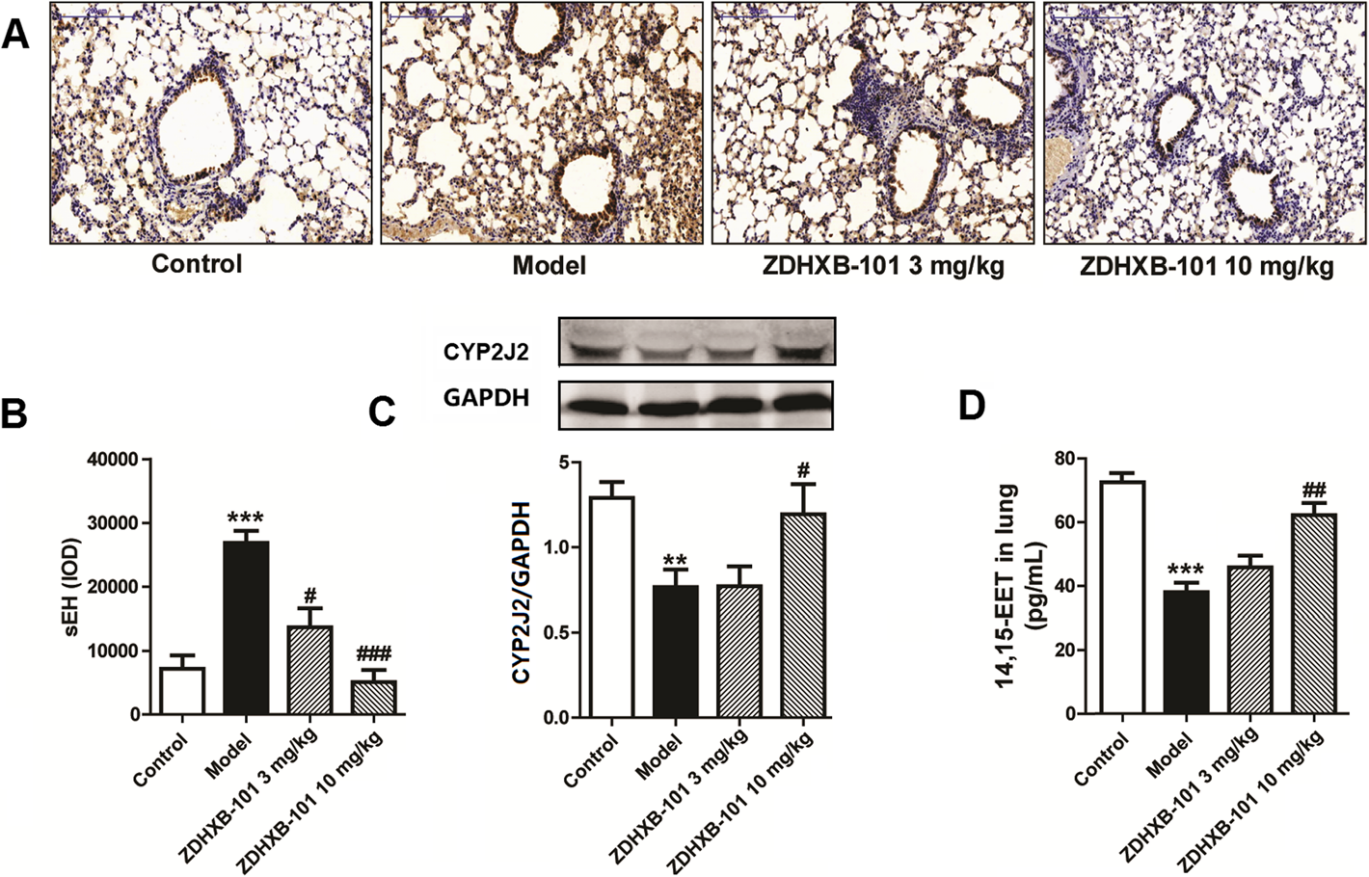
The effects of ZDHXB-101 on the OVA-induced expression of soluble epoxide hydrolase, CYP2J2 and 14,15-EETs. The protein level of soluble epoxide hydrolase (sEH) was assessed by immunohistochemistry (**a-b**), and the CYP2J2 and 14,15-EET levels were assessed by Western blot (**c**) and ELISA (**d**), respectively. All measurements were performed 24 h after the last challenge with treatment of ZDHXB-101 at doses of 3 mg/kg and 10 mg/kg. Scale bar: 100 μm. The data represent means ± S.D. Immunohistochemistry and ELISA assay (*n* = 10), Western blot (*n* = 6). ^***^p < 0.001 vs. the control group. ^#^*p* < 0.05, ^##^*p* < 0.01, and ^###^*p* < 0.001 vs. the model group

To verify the changes in the CYP450 pathway in the chronic asthma model, the expression of she and CYP2J2 and the levels of 14, 15-EETs were observed by immunohistochemical assay, Western blot and ELISA, respectively. The results are shown in Fig. 2. After the antigen challenge in sensitized mice, the expression of sEH protein increased by 2.73-fold, the expression of CYP2J2 decreased by 56.8%, and the levels of 14, 15-EETs dramatically decreased by 87.4% in the model group compared with the control group. These changes can be reversed by treatment with ZDHXB-101 10 mg/kg; however, 3 mg/kg ZDHXB-101 significantly reduced the expression of sEH, but it did not markedly affected the expression of CYP2J2 of the levels of the 14, 15-EETs (Fig. 2a-d).

### ZDHXB-101 protects against methacholine-induced AHR

**Fig. 3:**
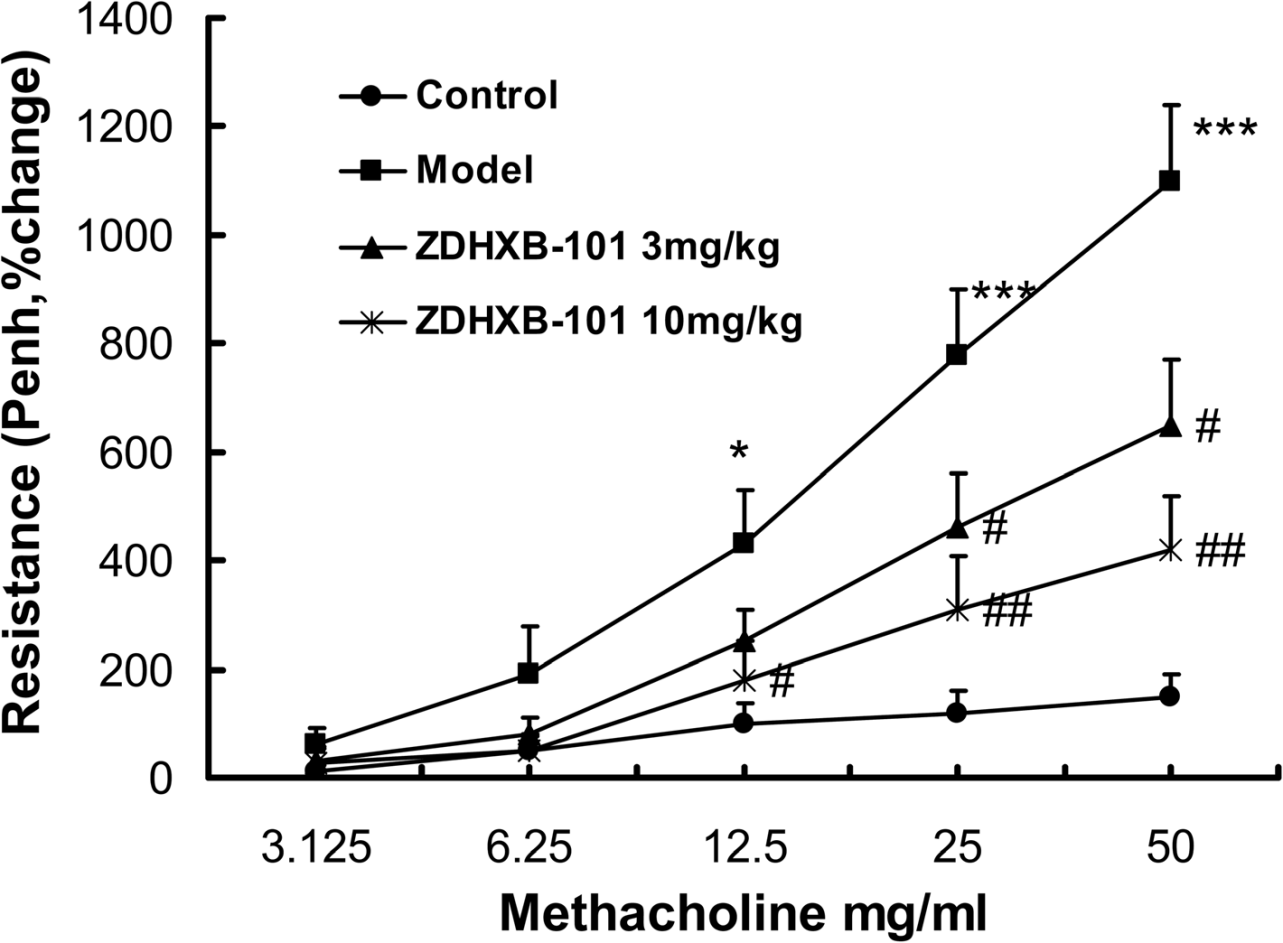
Effects of ZDHXB-101 on airway hyperresponsiveness. The lung function of mice in response to aerosolized methacholine (3.125–50 mg/mL) was measured 24 h after the last challenge with treatment of ZDHXB-101 at doses of 3 mg/kg and 10 mg/kg. Airway resistance was shown as Penh values. The results were expressed as percentage change in Penh above baseline. The data represent means ± S.D. (*n* = 10), ^*^p < 0.05 and ^***^p < 0.001 vs. the control group. ^#^*p* < 0.05 and ^##^*p* < 0.01 vs. the model group

To investigate the effect of ZDHXB-101 on AHR, airway resistance (Penh value) in response to methacholine (3.125–50 mg/ml) inhalation was measured using whole-body plethysmography. The airway resistance increased by 15-fold from baseline after exposure to 50 mg/ml Mch. The OVA-challenged mice showed markedly increased Penh values compared to the saline-challenged mice at 12.5 mg/ml to 50 mg/ml of Mch (*P* < 0.05, *P* < 0.001). The airway resistance increased 11-fold from baseline at the Mch 50 mg/ml concentration, which was significantly reduced by 32.7 and 56.4% at 3 mg/kg and 10 mg/kg ZDHXB-101, compared to the model group (*P* < 0.05~0.01) (Fig. 3).

### ZDHXB-101 inhibits inflammatory infiltration in lung tissues

**Fig. 4:**
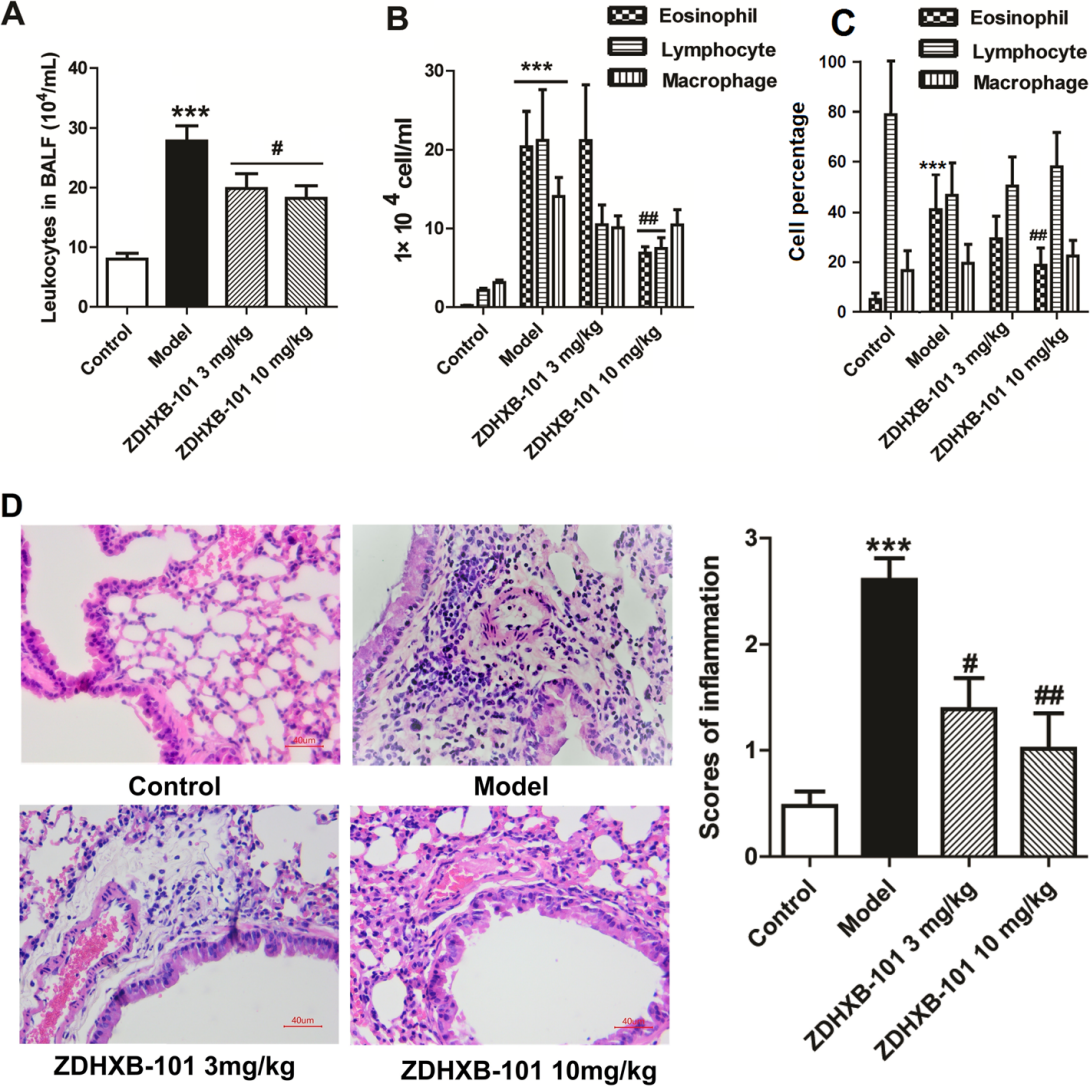
Effects of ZDHXB-101 on airway inflammation. **a** The total number of inflammatory cells in the BALF was counted, the (**b**) differential cells were determined in the BALF by Wright-Giemsa staining and cell classification was performed on a minimum of 200 cells to classify eosinophils, neutrophils, lymphocytes and macrophages 24 h after the final antigen challenge. **c** Lung sections were stained with H&E for the measurement of inflammatory cells infiltration in the peribronchiolar space. Inflammatory cell infiltration was graded based on the severity of inflammation (histogram). The data represent the means ± S.D. (*n* = 10). ^***^p < 0.001 vs. the control group. ^#^*p* < 0.05 and ^##^*p* < 0.01 vs. the model group. Scale bar: 40 μm

The number of inflammatory cells in the BALF was measured among different groups. As shown in Fig. 4a-b, the numbers of the total leucocytes, eosinophils, lymphocytes and macrophages were significantly higher in the model group than in the control group (*P* < 0.001). The percentage of eosinophils in the model group increased significantly (*P* < 0.001), whereas the percentage of lymphocytes decreased without a statistically significant difference compared with the model group. Treatment with 3 or 10 mg/kg ZDHXB-101 significantly inhibited the numbers of total leucocytes, eosinophils and lymphocytes, but not macrophages compared to the model group (*P* < 0.05~0.01). Treatment with 10 mg/kg ZDHXB-101 significantly inhibited the increase in the percentage of eosinophils (Fig. 4c).

A histological examination of lung sections from the model group showed a marked infiltration of eosinophil and lymphocytes cells in the peribronchiolar space, compared to control group, as shown in the H&E-stained lung sections (Fig. 4d). ZDHXB-101 clearly showed less inflammatory cell infiltration than model group, which showed typically airway inflammation that is characterized by the significant infiltration of eosinophils and lymphocytes in the peribronchiolar space (Fig. 4d).

### ZDHXB-101 alleviates the mRNA and protein levels of IL-13, IL-17 and MMP-9

**Fig. 5:**
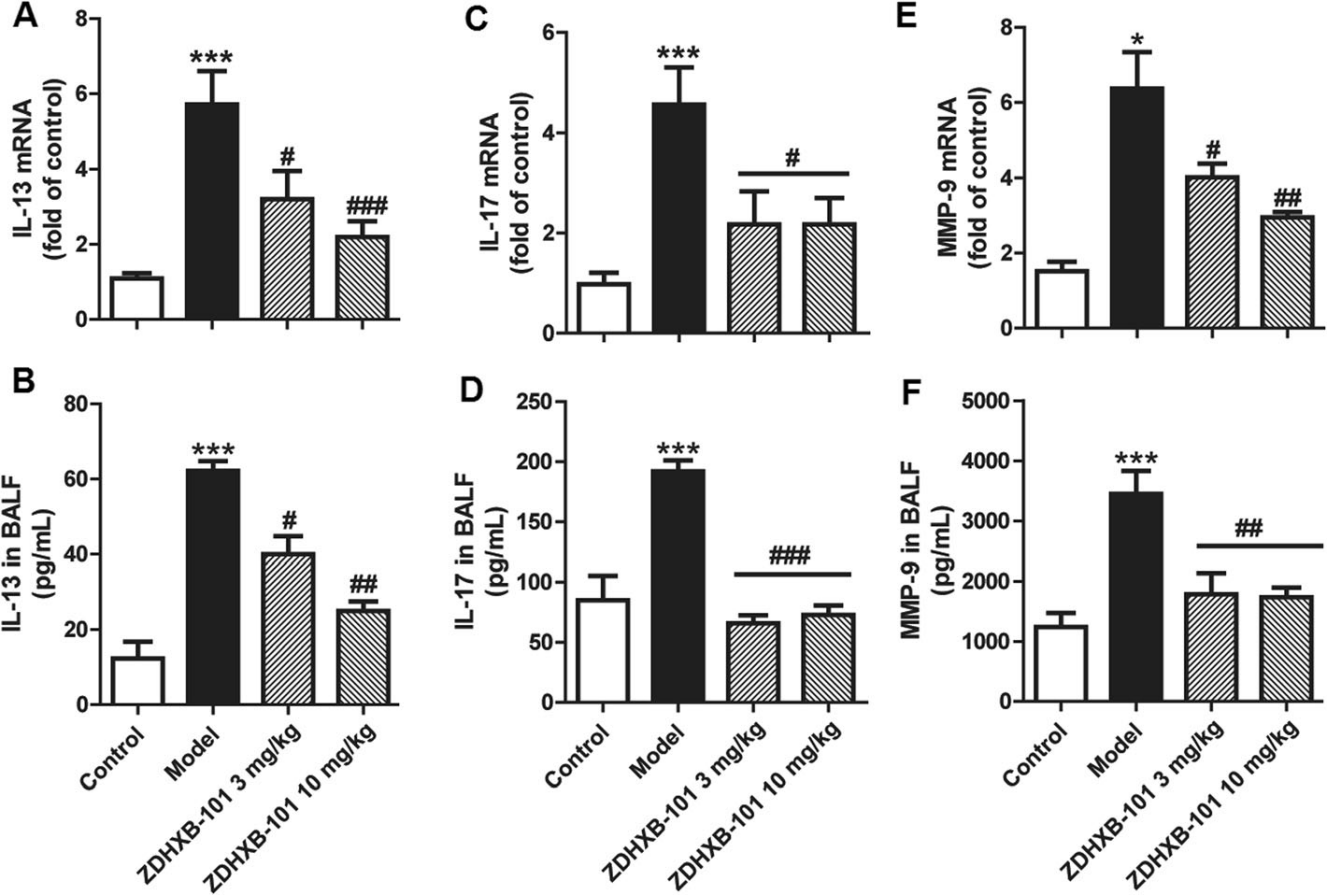
Effects of ZDHXB-101 on the expression of IL-13, IL-17 and MMP-9 in lung tissues. IL-13, IL-17 and MMP-9 mRNA and protein in the lung tissues were measured by q-PCR (**a**, **c**, **e**) and ELISA (**b**, **d**, **f**). The data represent means ± S.D. (*n* = 10), ^*^p < 0.05 and ^***^p < 0.001 vs. the control group. ^#^*p* < 0.05, ^##^*p* < 0.01, and ^###^*p* < 0.001 vs. the model group

To investigate the inhibitory effects of AUDA on the OVA-induced IL-13, IL-17 and MMP-9 levels, lung tissues and BALF were collected 24 h after the last challenge with AUDA treatment. IL-13, IL-17 and MMP-9 mRNA expression and protein production were measured using qRT-PCR and ELISA, respectively. As shown in Fig. 5, IL-13, IL-17 and MMP-9 mRNA expression in the lung tissues were significantly elevated in the model group compared to the control group (*P* < 0.05 or *P* < 0.001). Treatment with 3 or 10 mg/kg ZDHXB-101 markedly reduced the mRNA expression of IL-13, IL-17 and MMP-9 (*P* < 0.05 to *P* < 0.001) (Fig. 5a, c, e). We then measured protein levels and found that the protein levels of IL-13, IL-17 and MMP-9 were consistent with their changes in the mRNA expression (Fig. 5b, d, f).

### ZDHXB-101 improves airway remodeling markers

**Fig. 6:**
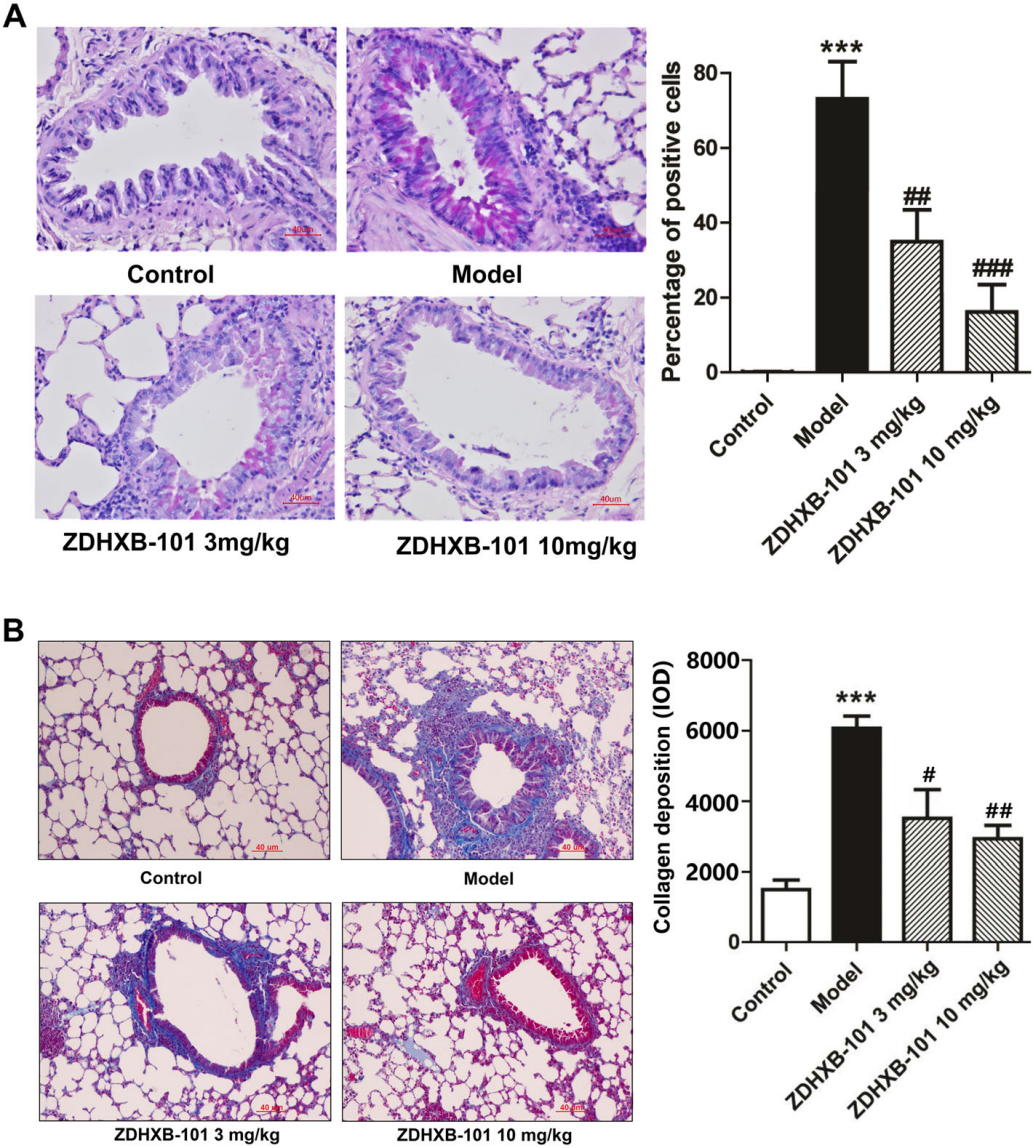
Effects of ZDHXB-101 on goblet cell hyperplasia and collagen deposition. **a** Lung sections were stained with PAS to assess goblet cell hyperplasia. PAS-positive and PAS-negative epithelial cells were counted, and the percentage of PAS-positive cells per bronchiole was calculated (histogram). **b** Lung sections were stained with Masson’s trichrome stains for the measurement of the subepithelial deposition of collagen and fibrosis. A Masson’s trichrome staining analysis of collagen deposition was conducted (histogram). The data represent means ± S.D. (*n* = 10), ^***^p < 0.001 vs. the control group. ^#^*p* < 0.05 and ^##^*p* < 0.01 vs. the model group. Scale bar: 40 μm

To evaluate the effects of ZDHXB-101 on OVA-induced goblet cell hyperplasia in the airway epithelium and collagen deposition around the bronchus, the cells of that were positive for PAS straining and the accumulations of collagen deposition that were indicated by Masson’s straining were determined. As shown in Fig. 6a and b, the percentage of PAS-positive cells in the model group was significantly higher than that in the control group (*P* < 0.001), but this increase was significantly weakened by 3 mg/kg and 10 mg/kg ZDHXB-101 (*P* < 0.01 and *P* < 0.001) (Fig. 6a). Similarly, the model group showed the emergence of thicker-walled alveoli with small diameters, thickened septa and accumulations of collagen deposition (blue staining) around the bronchus that were significantly different from the control group. Treatment with 3 mg/kg and 10 mg/kg ZDHXB-101 decreased these pathological changes in a dose-related fashion (Fig. 6b).

**Fig. 7:**
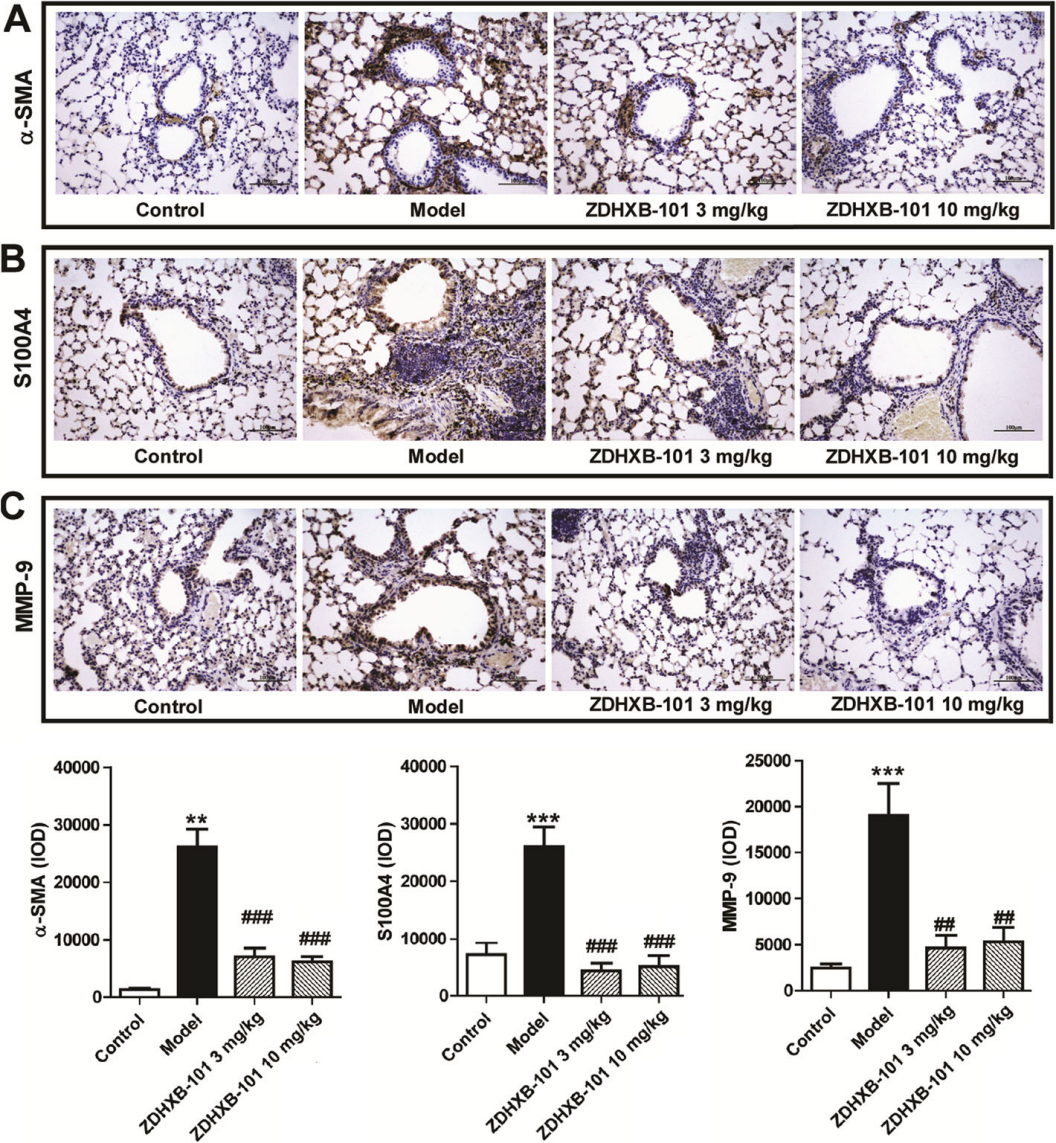
Effects of ZDHXB-101 on the overexpression of α-SMA, S100A4 and MMP-9. The representative pictures and paraffin sections were prepared and stained with immunohistochemistry to evaluate the protein expression of α-SMA, S100A4 and MMP-9in lung tissues. A semiquantitative analysis was performed. The data represent means ± S.D. (*n* = 10), ^**^p < 0.01 and ^***^p < 0.001 vs. the control group. ^##^*p* < 0.01and ^###^*p* < 0.001 vs. the model group. Scale bar: 100 μm

**Fig. 8:**
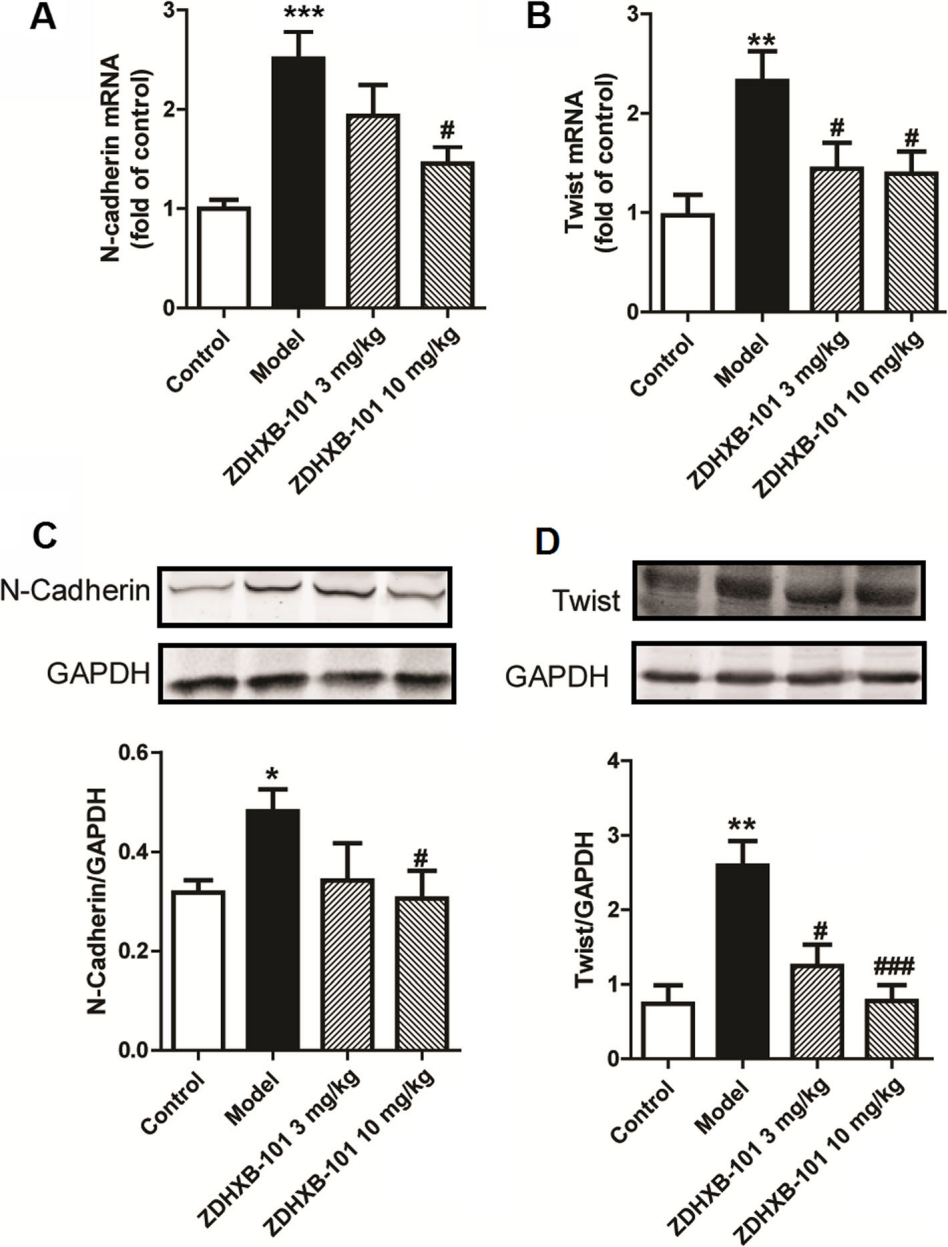
Effects of ZDHXB-101 on the expression of N-cadherin and Twist. The mRNA and protein expression of N-cadherin and Twist were by evaluated by q-PCR (**a-b**) and Western blot (**c-d**). The data represent means ± S.D. (*n* = 10), ^*^p < 0.05, ^**^p < 0.01 and ^***^p < 0.001 vs. the control group. ^#^*p* < 0.05 and ^###^*p* < 0.001 vs. the model group

To investigate the effects of ZDHXB-101 on the OVA-induced protein expression of EMT-related markers, lung tissues were collected 24 h after the final antigen challenge. Immunohistochemistry was employed to investigate the expression of the α-SMA, S100A4 and MMP-9 proteins in the lung tissues. As shown in Fig. 7, the expression of α-SMA, S100A4 and MMP-9 proteins in the model group was significantly higher than that in the control group, but ZDHXB-101 treatment markedly improved these changes. In addition, the N-cadherin and Twist mRNA expression and protein levels were examined using qPCR and Western Blot, respectively. We found an increase in both N-cadherin and Twist mRNA and proteins, and the protein levels were consistent with their changes in the mRNA expression. However, treatment with 3 or 10 mg/kg ZDHXB-101 significantly inhibited the changes in N-cadherin and Twist mRNA and protein in the lung tissues compared to the model group (Fig. 8a-d).

### ZDHXB-101 downregulates the activation of MAPKs and STAT3 signaling

**Fig. 9:**
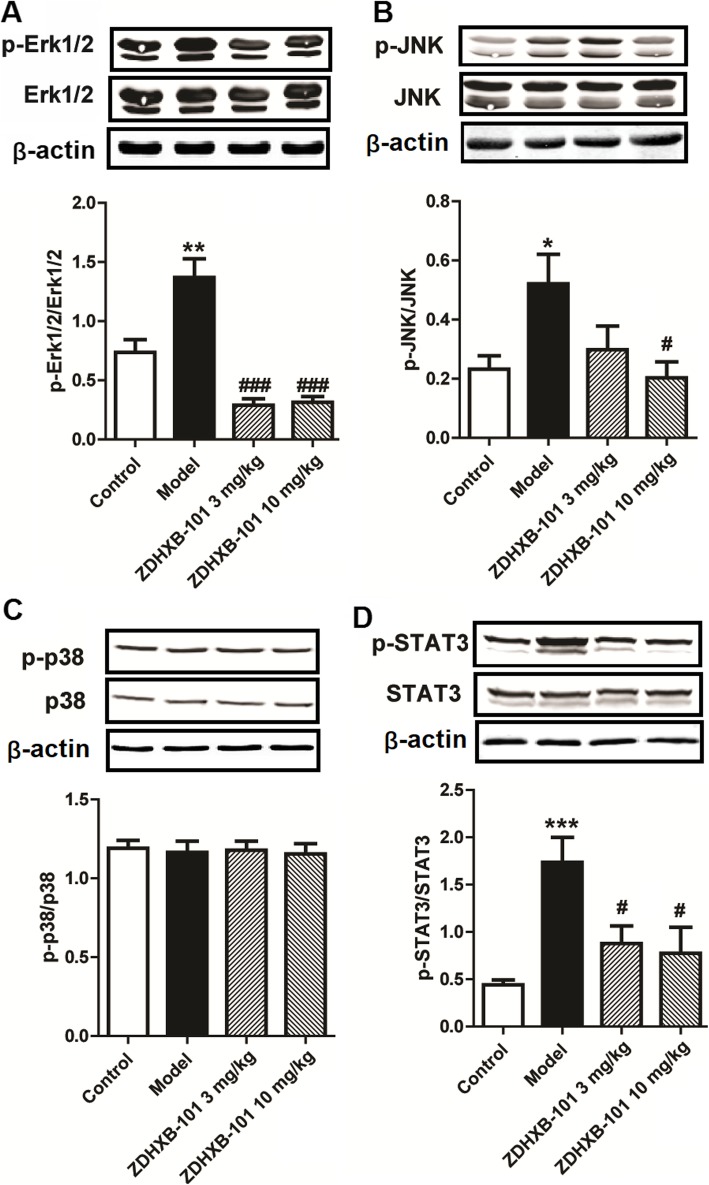
Effects of ZDHXB-101 on the activation of the Erk1/2, JNK MAPK and STAT3 pathways. The mouse lung tissues from different treatment groups were harvested and the phosphorylation of Erk1/2 (**a**), JNK (**b**), p38 (**c**) and STAT3 (**d**) was measured by Western blot. The data represent means ± S.D. (*n* = 8), ^*^p < 0.05, ^**^p < 0.01 and ^***^p < 0.001 vs. the control group. ^#^*p* < 0.05 and ^###^*p* < 0.001 vs. the model group

To assess the action mechanism of ZDHXB-101 against airway remodeling and AHR, we analyzed a number of related proteins in the MAPKs and STAT3 signaling pathways by Western blot. As illustrated in Fig. 9a, b, d, the levels of phosphorylated Erk1/2, JNK and phosphorylated STAT3 proteins were significantly higher in the model group than those in the control group. These changes were inverted by ZDHXB-101 treatment. However, treatment had no effects on the phosphorylation of p38 in vivo (Fig. 9c). These results demonstrated that ZDHXB-101 may act through the Erk1/2, JNK MAPKs and STAT3 signaling pathways.

## Discussion

Severe asthma is characterized by airway remodeling and AHR and is associated with aberrant metabolism of arachidonic acid. A clinical study in the asthmatic patients has demonstrated that the levels of EETs were decreased by oxidative stress and sEH activity. Inhibitors of sEH increased the EETs that mediated antiphlogistic actions, suggesting a new therapeutic approach for severe asthma [23]. In experimental studies, inhibitors of sEH have been shown to reduce lung injury caused by lipopolysaccharide [24, 25], pulmonary fibrosis induced by bleomycin [15], asthma caused by OVA [12], and airway inflammation induced by cigarette smoke [26, 27]. EETs can relax human airway smooth muscles and directly activate reconstituted KCa channels [28] and are potent modulators of the hyperreactivity triggered by TNF-α in human airway smooth muscle cells [29]. In previous studies, we also found that EETs attenuate cigarette smoke extract-induced interleukin-8 production in bronchial epithelial cells [30] and alleviate ox-LDL-induced inflammation by inhibiting LOX-1 receptor expression in rat pulmonary arterial endothelial cells [31, 32]. In this study, we explored the outcome and mechanism of inhibiting sEH elevation and EETs in airway remodeling and hyperresponsiveness in chronic asthma. ZDHXB-101 is a natural product and a honokiol structural derivative. In our preliminary study, it has been proven to inhibit the initial activity on human recombinant sEH not only had significantly increased EET levels of human bronchial epithelial cells, where it but also reversed the effect of a TGF-β1-induced an increase in sEH protein expression compared with the vehicle group (Additional file 1). However, it is not clear whether, as a new sEH inhibitor, ZDHXB-101 is effective in airway remodeling and AHR in a chronic asthmatic model. EETs are the downstream metabolites of CYP2J or CYP2C and could be hydrolyzed by sEH to less active dihydroxyeicosatrienoic acids [33]. In this study, we found that CYP2J2 protein expression and the 14,15-EETs levels were significantly suppressed in lung tissues of the OVA mouse model. The ZDHXB-101-treated group had significantly increased CYP2J2 expression and 14,15-EETs levels compared with the model group. Moreover, the asthmatic group showed an increase in sEH protein expression that was reversed by ZDHXB-101 treatment. These results demonstrate that the CYP pathway is involved in airway remodeling that is triggered in the chronic asthmatic model. This is in agreement with a previous report demonstrating that an sEH inhibitor attenuates inflammation and airway hyperresponsiveness by inhibiting sEH and increasing the levels of EETs in mice [14, 15].

The airway remodeling in the asthmatic model is the primary abnormal response to persistent airway inflammation. In fact, airway remodeling in asthma has been shown to be the result of a chronic inflammatory response entailing on the one hand permanent airway tissue destruction and on the other hand chronic tissue repair. Thus, chronic airway inflammation can be described as the major force driving the processes leading to most aspects of airway remodeling [34]. This inflammation theory is mainly supported by the finding that steroid treatment in asthmatic patients does not only reduce airway inflammation but also has beneficial effects on airway remodeling [8, 9]. Infiltrating cells like T helper (Th) cells, eosinophils, neutrophils and mast cells interact with the resident cells of the airways such as fibroblasts, smooth muscle cells, neuronal cells, epithelial cells and endothelial cells by the releasing plethora of cytokines, enzymes, metabolites and growth factors creating a signaling environment that under chronic conditions results in airway remodeling. In this study, we found that after sensitization and challenge with OVA, the number of total leucocytes, eosinophils and lymphocytes in the BALF were evidently higher than those in the control group and that ZDHXB-101 treatment noticeably prevented inflammatory cell infiltration into the airways, as shown by a significant decrease in total cell counts, eosinophils and lymphocytes in a dose-dependent manner. The histological examination of H&E-stained lung tissues revealed the infiltration of inflammatory cells, such as lymphocytes and eosinophils after the OVA-challenge. Thus, the role of ZDHBX-101 in inhibiting airway remodeling is at least partially related to its anti-inflammatory effects. Further anti-inflammatory evidence could also be obtained from the effect of ZDHBX-101 in reducing the mRNA and protein expression of the proinflammatory cytokines IL-13 and IL-17.

T helper cells in allergic bronchial tissues, especially Th2 cells, orchestrate the allergic inflammatory response by releasing a characteristic array of cytokines including IL-4, IL-5 and IL-13 [35]. However, whether they have a direct impact on airway remodeling is still a matter of debate. As already mentioned, the effects of IL-4, IL-5 and IL-9 to promote airway remodeling are at least partly dependent on IL-13 [36]. Only IL-13 has been identified to have profound effects on airway structural cells [36, 37]. In mice, IL-13 induces mucin expression and mucus metaplasia in both airway epithelial cells and submucosal glands [38] and also plays a key role in goblet cell hyper/metaplasia in humans [39]. Furthermore, IL-13 induces the release of the profibrotic TGF-β by epithelial cells [37]. However, whether Th17 cells directly contribute to airway remodeling is part of an ongoing discussion. In a mouse model of chronic experimental asthma, the absence of Th17 cells resulted in diminished airway remodeling as demonstrated by reduced staining of collagen fibers and α-smooth muscle actin, although allergic airway inflammation remained unaltered [40]. Lu et al. [41] also used a mouse model of chronic experimental asthma and correlated the progressively increasing levels of Th17 cells and IL-17A with peribronchial microvessel density. The neutralization of IL-17 abrogated these signs of airway vascular remodeling. Recent gene expression analyses of the endobronchial tissue have revealed three major patient clusters: The Th2-high, Th17-high, and Th2/17-low. Th2-high and Th17-high patterns were mutually exclusive in individual patient samples, and their gene signatures were inversely correlated and differentially regulated by IL-13 and IL-17A. The neutralization of IL-4 and/or IL-13 resulted in increased Th17 cells and neutrophilic inflammation in the lung. However, the neutralization of IL-13 and IL-17 protected mice from eosinophilia, mucus hyperplasia, and airway hyperreactivity and abolished the neutrophilic inflammation, suggesting that combination therapies targeting both pathways may maximize therapeutic efficacy across a patient population comprising both TH2 and TH17 endotypes [42]. Therefore, in this experiment we examined the two cytokines IL-13 and IL-17. The results showed that ZDHXB-101 inhibited both proinflammatory cytokines.

Another important characteristic of persistent airflow obstruction in asthma is airway remodeling, including GCM, EMT, subepithelial collagen deposition, airway smooth muscle hyperplasia, and increased vascularity [5, 6]. Increasing evidence suggests the involvement of EMT in the bronchial remodeling of asthmatic patients [43–46]. In particular, the epithelium is increasingly gaining significant importance considering its potential as a source and target of inflammatory mediators, extracellular matrix components and growth factors. Our studies showed airway remodeling and inflammation after long-term exposure to an antigen in a chronic asthmatic model over a period of 12 weeks. Airway remodeling and the expression of EMT-related markers such as expression of N-cadherin, α-SMA, S100A4, and Twist were increased after exposure to the antigen. This finding is in agreement with previous reports in which the airway epithelial cells of a transgenic murine model were shown to express the lac-Z reporter gene. After 5 days of exposure to house dust mite allergen, the epithelial cells underwent EMT, coexpressed the protein S100A4 and accumulated in the smooth muscle. Other epithelial cells co-expressing vimentin were found in the subepithelial region. These results suggest that EMT can be implicated in the airway remodeling process that is associated with asthma [16, 45, 46]. In addition, in this study, the indicators that are associated with airway remodeling such as bronchial epithelial goblet cell metaplasia, collagen deposition, and the overexpression of the α-SMA, S100A4 and MMP-9 proteins, were also found by histological and immunohistochemical examinations. The change of AHR pulmonary function as a comprehensive result of airway remodeling was one of clinical manifestations of the chronic asthmatic model. Our results showed that ZDHXB-101 decreased airway remodeling-related markers such as goblet cell metaplasia, collagen deposition, overexpression of N-cadherin, α-SMA, S100A4, Twist, and MMP-9, thereby improving AHR. Thus, it can be seen that the effect of ZDHXB-101 on EMT in lung epithelial cells is similar to that of honokiol on EMT in renal epithelial cells [47, 48] and mammary epithelial cells [49].

To further dissect the molecular mechanisms underlying the effect of ZDHXB-101 on airway remodeling and inflammation, we focused on the MAPKs and STAT3 pathways. The MAPK family is fundamental in regulating multiple cell functions such as cytokine expression, proliferation, and apoptosis. The activation of MAPK and STAT3 in the airways drives a majority of the allergen-induced inflammatory response and is a significant contributor to the structural remodeling of the airway wall [16, 50, 51]. Its increased activation has been demonstrated in the lungs after allergen challenge and in airway epithelial cells, lung fibroblasts and airway smooth muscle cells. Therefore, MAPK has emerged as a promising molecular target for the treatment of asthma [50]. The results in this study indicated that lung tissues in the chronic asthmatic model showed significantly increased the phosphorylation of Erk1/2 and JNK protein expression in the lung tissues, whereas ZDHXB-101 markedly suppressed Erk1/2 and JNK activity in an OVA-induced model of asthma. However, it had no effects on the phosphorylation of p38. STAT3 was been shown earlier to regulate the allergic response in asthma. Specifically, epithelial STAT3 was identified as a critical regulator of allergen-induced inflammation and AHR in a murine model of asthma [52]. Here we observed a clear phosphorylation of STAT3 in response to OVA, and ZDHXB-101 inhibited the phosphorylation of STAT3 in an OVA-induced allergy model of asthma. These findings suggested that the therapeutic effects of ZDHXB-101 were correlated with the MAPKs and STAT3 signaling pathways.

## Conclusions

We present the first study demonstrating that ZDHXB-101 effectively reduced airway remodeling, inflammation and AHR by inhibiting sEH and increasing the levels of EETs in the chronic asthmatic model. In addition, the inhibitory effects of ZDHXB-101 were associated with regulation of the phosphorylation of Erk1/2, JNK and STAT3. These findings provide effective evidence that ZDHXB-101 may be a potent therapeutic agent for asthma.

## Additional file

**Additional file 1: Figure S1.** Chemical structure of ZDHXB-101. **Figure S2.** Effects of ZDHXB-101 on cell proliferation and activity in 16HBE cells, inhibits TGFβ1-induced increase in soluble epoxide hydrolase (sEH) expression and decrease in 14, 15-EETs levels. (A and D) cell proliferation; (C) cell activity; (D and E) sEH protein expression; (F) 14, 15-EETs level. 16HBE cell was treated with the indicated concentrations (5, 10, 20 μM) of ZDHXB-101or AUDA for 24–72 h. The viability levels of 16HBE cells at the logarithmic phase were determined using the MTT assay (*n* = 6 per group). The lactate dehydrogenase (LDH) levels were determined using ELISA assay (*n* = 6 per group). (D and E) The sEH expression of 16HBE cells were induced with the indicated concentrations (1.25–10 μM) of TGFβ1 for 24 h. The protein levels of sEH were assessed by western blot. The 14, 15-EETs levels were determined using ELISA assay (*n* = 6 per group). The data represent mean ± S.E.M. from 4 independent experiments, *p < 0.05, **p < 0.01 and ***p <0.001 compared with the untreated group. #*p* < 0.05 indicates significant differences between the TGFβ1 group and the TGFβ1 + AUDA group.

## Data Availability

The analyzed datasets generated during the study are available from the corresponding author on reasonable request.

## Abbreviations

AHR: Airway hyperresponsiveness
AUDA: A soluble epoxide hydrolase inhibitor
BALF: Bronchoalveolar lavage fluid
CYP: Cytochrome P450
EET: Epoxyeicosatrienoic acid
ELISA: Enzyme-linked immunosorbent assay
EMT: Epithelial-to-mesenchymal transition
Erk1/2: Extracellular regulated protein kinases 1/2
H&E: Hematoxylin and eosin
IL: Interleukin
JNK: c-Jun N-terminal kinases
MAPK: Mitogen-activated protein kinase
MMP-9: Matrix metalloproteinase 9
OVA: Ovalbumin
PAS: Periodic acid-Schiff
Penh: Enhanced pause
qPCR: Quantitative polymerase chain reaction
sEH: Soluble epoxide hydrolase
sHE: Soluble epoxide hydrolase
STAT3: Signal transducer and activator of transcription-3
WBP: Whole-body plethysmography
α-SMA: α-smooth muscle actin

## References

[1] Saglani S, Lloyd CM (2015). Novel concepts in airway inflammation and remodelling in asthma. Eur Respir J.

[2] Global Initiative for Asthma (GINA), 2019 (2019). Global strategy for asthma management and prevention.

[3] Dunican EM, Elicker BM, Gierada DS, Nagle SK, Schiebler ML, Newell JD, Raymond WW, Lachowicz-Scroggins ME, Di Maio S, Hoffman EA (2018). Mucus plugs in patients with asthma linked to eosinophilia and airflow obstruction. J Clin Invest.

[4] Fehrenbach H, Wagner C, Wegmann M (2017). Airway remodeling in asthma: what really matters. Cell Tissue Res.

[5] Carpaij OA, Burgess JK, Kerstjens HAM, Nawijn MC, van den Berge M (2019). A review on the pathophysiology of asthma remission. Pharmacol Ther.

[6] Malmström K, Lohi J, Sajantila A, Jahnsen FL, Kajosaari M, Sarna S, Mäkelä MJ (2017). Immunohistology and remodeling in fatal pediatric and adolescent asthma. Respir Res.

[7] Tillie-Leblond I, de Blic J, Jaubert F, Wallaert B, Scheinmann P, Gosset P (2008). Airway remodeling is correlated with obstruction in children with severe asthma. Allergy..

[8] Nayak AP (2019). Glucocorticoids and airway smooth muscle: a few more answers, still more questions. Am J Respir Cell Mol Biol.

[9] Roth M, Johnson PR, Borger P, Bihl MP, Rüdiger JJ, King GG, Ge Q, Hostettler K, Burgess JK, Black JL, Tamm M (2004). Dysfunctional interaction of C/EBPalpha and the glucocorticoid receptor in asthmatic bronchial smooth-muscle cells. N Engl J Med.

[10] Chakir J, Haj-Salem I, Gras D, Joubert P, Beaudoin ÈL, Biardel S, Lampron N, Martel S, Chanez P, Boulet LP, Laviolette M (2015). Effects of bronchial thermoplasty on airway smooth muscle and collagen deposition in asthma. Ann Am Thorac Soc.

[11] Aliwarga T, Evangelista EA, Sotoodehnia N, Lemaitre RN, Totah RA (2018). Regulation of CYP2J2 and EET levels in cardiac disease and diabetes. Int J Mol Sci.

[12] Zhou C, Huang J, Chen J, Lai J, Zhu F, Xu X, Wang DW (2016). CYP2J2-derived EETs attenuated angiotensin II-induced adventitial remodeling via reduced inflammatory response. Cell Physiol Biochem.

[13] Jamieson KL, Endo T, Darwesh AM, Samokhvalov V, Seubert JM (2017). Cytochrome P450-derived eicosanoids and heart function. Pharmacol Ther.

[14] Simpkins AN, Rudic RD, Roy S, Tsai HJ, Hammock BD, Imig JD (2010). Soluble epoxide hydrolase inhibition modulates vascular remodeling. Am J Physiol Heart Circ Physiol.

[15] Yang J, Bratt J, Franzi L, Liu JY, Zhang G, Zeki AA, Vogel CF, Williams K, Dong H, Lin Y, Hwang SH, Kenyon NJ, Hammock BD (2015). Soluble epoxide hydrolase inhibitor attenuates inflammation and airway hyperresponsiveness in mice. Am J Respir Cell Mol Biol.

[16] Pu Q, Zhao Y, Sun Y, Huang T, Lin P, Zhou C, Qin S, Singh BB, Wu M (2019). TRPC1 intensifies house dust mite-induced airway remodeling by facilitating epithelial-to-mesenchymal transition and STAT3/NF-κB signaling. FASEB J.

[17] Schuliga M (2015). NF-kappaB signaling in chronic inflammatory airway disease. Biomolecules.

[18] Vale K (2016). Targeting the JAK-STAT pathway in the treatment of 'Th2-high' severe asthma. Future Med Chem.

[19] Glaab T, Taube C, Braun A, Mitzner W (2007). Invasive and noninvasive methods for studying pulmonary function in mice. Respir Res.

[20] Hoymann HG (2007). Invasive and noninvasive lung function measurements in rodents. J Pharmacol Toxicol Methods.

[21] Cao R, Dong XW, Jiang JX, Yan XF, He JS, Deng YM, Li FF, Bao MJ, Xie YC, Chen XP, Xie QM (2011). M(3) muscarinic receptor antagonist bencycloquidium bromide attenuates allergic airway inflammation, hyperresponsiveness and remodeling in mice. Eur J Pharmacol.

[22] Livak KJ, Schmittgen TD (2001). Analysis of relative gene expression data using real-time quantitative PCR and the 2(−Delta Delta C(T)) method. Methods..

[23] Ono E, Dutile S, Kazani S, Wechsler ME, Yang J, Hammock BD, Douda DN, Tabet Y, Khaddaj-Mallat R, Sirois M (2014). Lipoxin generation is related to soluble epoxide hydrolase activity in severe asthma. Am J Respir Crit Care Med.

[24] Zhou Y, Liu T, Duan JX, Li P, Sun GY, Liu YP, Zhang J, Dong L, Lee KSS, Hammock BD (2017). Soluble epoxide hydrolase inhibitor attenuates lipopolysaccharide-induced acute lung injury and improves survival in mice. Shock.

[25] Tao W, Li PS, Yang LQ, Ma YB (2016). Effects of a soluble epoxide hydrolase inhibitor on lipopolysaccharide-induced acute lung injury in mice. PLoS One.

[26] Wang L, Yang J, Guo L, Uyeminami D, Dong H, Hammock BD, Pinkerton KE (2012). Use of a soluble epoxide hydrolase inhibitor in smoke-induced chronic obstructive pulmonary disease. Am J Respir Cell Mol Biol.

[27] Smith KR, Pinkerton KE, Watanabe T, Pedersen TL, Ma SJ, Hammock BD (2005). Attenuation of tobacco smoke-induced lung inflammation by treatment with a soluble epoxide hydrolase inhibitor. Proc Natl Acad Sci U S A.

[28] Dumoulin M, Salvail D, Gaudreault SB, Cadieux A, Rousseau E (1998). Epoxyeicosatrienoic acids relax airway smooth muscles and directly activate reconstituted KCa channels. Am J Phys.

[29] Morin C, Sirois M, Echave V, Gomes MM, Rousseau E (2008). EET displays anti-inflammatory effects in TNF-alpha stimulated human bronchi: putative role of CPI-17. Am J Respir Cell Mol Biol.

[30] Ma WJ, Sun YH, Jiang JX, Dong XW, Zhou JY, Xie QM (2015). Epoxyeicosatrienoic acids attenuate cigarette smoke extract-induced interleukin-8 production in bronchial epithelial cells. Prostaglandins Leukot Essent Fat Acids.

[31] Jiang JX, Zhang SJ, Liu YN, Lin XX, Sun YH, Shen HJ, Yan XF, Xie QM (2014). EETs alleviate ox-LDL-induced inflammation by inhibiting LOX-1 receptor expression in rat pulmonary arterial endothelial cells. Eur J Pharmacol.

[32] Jiang JX, Zhang SJ, Xiong YK, Jia YL, Sun YH, Lin XX, Shen HJ, Xie QM, Yan XF (2015). EETs attenuate ox-LDL-induced LTB4 production and activity by inhibiting p38 MAPK phosphorylation and 5-LO/BLT1 receptor expression in rat pulmonary arterial endothelial cells. PLoS One.

[33] Shahabi P, Siest G, Meyer UA, Visvikis-Siest S (2014). Human cytochrome P450 epoxygenases: variability in expression and role in inflammation-related disorders. Pharmacol Ther.

[34] Zhou-Suckow Z, Duerr J, Hagner M, Agrawal R, Mall MA (2017). Airway mucus, inflammation and remodeling: emerging links in the pathogenesis of chronic lung diseases. Cell Tissue Res.

[35] McGee HS, Agrawal DK (2006). TH2 cells in the pathogenesis of airway remodeling: regulatory T cells a plausible panacea for asthma. Immunol Res.

[36] Ingram JL, Kraft M (2012). IL-13 in asthma and allergic disease: asthma phenotypes and targeted therapies. J Allergy Clin Immunol.

[37] Malavia NK, Mih JD, Raub CB, Dinh BT, George SC (2008). IL-13 induces a bronchial epithelial phenotype that is profibrotic. Respir Res.

[38] Therien AG, Bernier V, Weicker S, Tawa P, Falgueyret JP, Mathieu MC, Honsberger J, Pomerleau V, Robichaud A, Stocco R (2008). Adenovirus IL-13-induced airway disease in mice: a corticosteroid-resistant model of severe asthma. Am J Respir Cell Mol Biol.

[39] Wang X, Li Y, Luo D, Wang X, Zhang Y, Liu Z, Zhong N, Wu M, Li G (2017). Lyn regulates mucus secretion and MUC5AC via the STAT6 signaling pathway during allergic airway inflammation. Sci Rep.

[40] Zhao J, Lloyd CM, Noble A (2013). Th17 responses in chronic allergic airway inflammation abrogate regulatory T-cell-mediated tolerance and contribute to airway remodeling. Mucosal Immunol.

[41] Lu S, Li H, Gao R, Gao X, Xu F, Wang Q, Lu G, Xia D, Zhou J (2015). IL-17A, but not IL-17F, is indispensable for airway vascular remodeling induced by exaggerated Th17 cell responses in prolonged ovalbumin-challenged mice. J Immunol.

[42] Choy DF, Hart KM, Borthwick LA, Shikotra A, Nagarkar DR, Siddiqui S, Jia G, Ohri CM, Doran E, Vannella KM (2015). TH2 and TH17 inflammatory pathways are reciprocally regulated in asthma. Sci Transl Med.

[43] Ijaz T, Pazdrak K, Kalita M, Konig R, Choudhary S, Tian B, Boldogh I, Brasier AR (2014). Systems biology approaches to understanding epithelial Mesenchymal transition (EMT) in mucosal remodeling and signaling in asthma. World Allergy Organ J.

[44] Post S, Heijink IH, Hesse L, Koo HK, Shaheen F, Fouadi M, Kuchibhotla VNS, Lambrecht BN, Van Oosterhout AJM, Hackett TL, Nawijn MC (2018). Characterization of a lung epithelium specific E-cadherin knock-out model: implications for obstructive lung pathology. Sci Rep.

[45] Fischer KD, Hall SC, Agrawal DK (2016). Vitamin D supplementation reduces induction of epithelial-mesenchymal transition in allergen sensitized and challenged mice. PLoS One.

[46] Johnson JR, Roos A, Berg T, Nord M, Fuxe J (2011). Chronic respiratory aeroallergen exposure in mice induces epithelial mesenchymal transition in the large airways. PLoS One.

[47] Chiang CK, Sheu ML, Lin YW, Wu CT, Yang CC, Chen MW, Hung KY, Wu KD, Liu SH (2011). Honokiol ameliorates renal fibrosis by inhibiting extracellular matrix and pro-inflammatory factors in vivo and in vitro. Br J Pharmacol.

[48] Li W, Wang Q, Su Q, Ma D, An C, Ma L, Liang H (2014). Honokiol suppresses renal cancer cells' metastasis via dual-blocking epithelial-mesenchymal transition and cancer stem cell properties through modulating miR-141/ZEB2 signaling. Mol Cell.

[49] Avtanski DB, Nagalingam A, Bonner MY, Arbiser JL, Saxena NK, Sharma D (2014). Honokiol inhibits epithelial-mesenchymal transition in breast cancer cells by targeting signal transducer and activator of transcription 3/Zeb1/E-cadherin axis. Mol Oncol.

[50] Zhang Y, Cardell LO, Edvinsson L, Xu CB (2013). MAPK/NF-κB-dependent upregulation of kinin receptors mediates airway hyperreactivity: a new perspective for the treatment. Pharmacol Res.

[51] Zhang Y, Li S, Huang S, Cao L, Liu T, Zhao J, Wu J, Wang J, Cao L, Xu J, Dong L (2019). IL33/ST2 contributes to airway remodeling via p-JNK MAPK/STAT3 signaling pathway in OVA-induced allergic airway inflammation in mice. Exp Lung Res.

[52] Simeone-Penney MC, Severgnini M, Tu P, Homer RJ, Mariani TJ, Cohn L, Simon AR (2007). Airway epithelial STAT3 is required for allergic inflammation in a murine model of asthma. J Immunol.

