# Supplementary material for: ZDHXB-101 (3′,5-Diallyl-2, 4′-dihydroxy-[1,1′-biphen-yl]-3,5′-dicarbaldehyde) protects against airway remodeling and hyperresponsiveness via inhibiting both the activation of the mitogen-activated protein kinase and the signal transducer and activator of transcription-3 signaling pathways
Source: Respir Res. 2020 Jan 13;21:22. doi: 10.1186/s12931-020-1281-x (PMC6958776; doi:10.1186/s12931-020-1281-x)
Supplement: Supplementary file 1 — Additional file 1: Figure S1. Chemical structure of ZDHXB-101. Figure S2. Effects of ZDHXB-101 on cell proliferation and activity in 16HBE cells, inhibits TGFβ1-induced increase in soluble epoxide hydrolase (sEH) expression and decrease in 14, 15-EETs levels. (A and D) cell proliferation; (C) cell activity; (D and E) sEH protein expression; (F) 14, 15-EETs level. 16HBE cell was treated with the indicated concentrations (5, 10, 20 μM) of ZDHXB-101or AUDA for 24–72 h. The viability levels of 16HBE cells at the logarithmic phase were determined using the MTT assay (n = 6 per group). The lactate dehydrogenase (LDH) levels were determined using ELISA assay (n = 6 per group). (D and E) The sEH expression of 16HBE cells were induced with the indicated concentrations (1.25–10 μM) of TGFβ1 for 24 h. The protein levels of sEH were assessed by western blot. The 14, 15-EETs levels were determined using ELISA assay (n = 6 per group). The data represent mean ± S.E.M. from 4 independent experiments, *p < 0.05, **p < 0.01 and ***p <0.001 compared with the untreated group. #p < 0.05 indicates significant differences between the TGFβ1 group and the TGFβ1 + AUDA group. [file 12931_2020_1281_MOESM1_ESM.docx]

**Supplementary data**

**ZDHXB-101 (3’,5-Diallyl-2, 4’-dihydroxy-[1,1’-biphen-yl]-3,5’-dicarbaldehyde)** **protects against airway remodeling and hyperresponsiveness via inhibiting both the activation of** **the mitogen-activated protein kinase and** **the signal transducer and activator of transcription-3 signaling pathways**

Jun-xia Jiang^a,b,1^, Hui-juan Shen^a,b,1^, Yan Guan^b,c^, Yong-liang Jia^b^, Jian Shen^b^, Qi Liu^b^, Qiang-min Xie^b,*^, Xiao-feng Yan^a,*^

^a^ The Second Affiliated Hospital, Zhejiang University School of Medicine, Hangzhou, China, 310009.

^b^ Zhejiang Respiratory Drugs Research Laboratory of State Food and Drug Administration of China, Zhejiang University School of Medicine, Hangzhou, China, 310058.

^c.^Affiliated Sir Run Run Shaw Hospital, Zhejiang University School of Medicine, Hangzhou, China.

**Additional file**


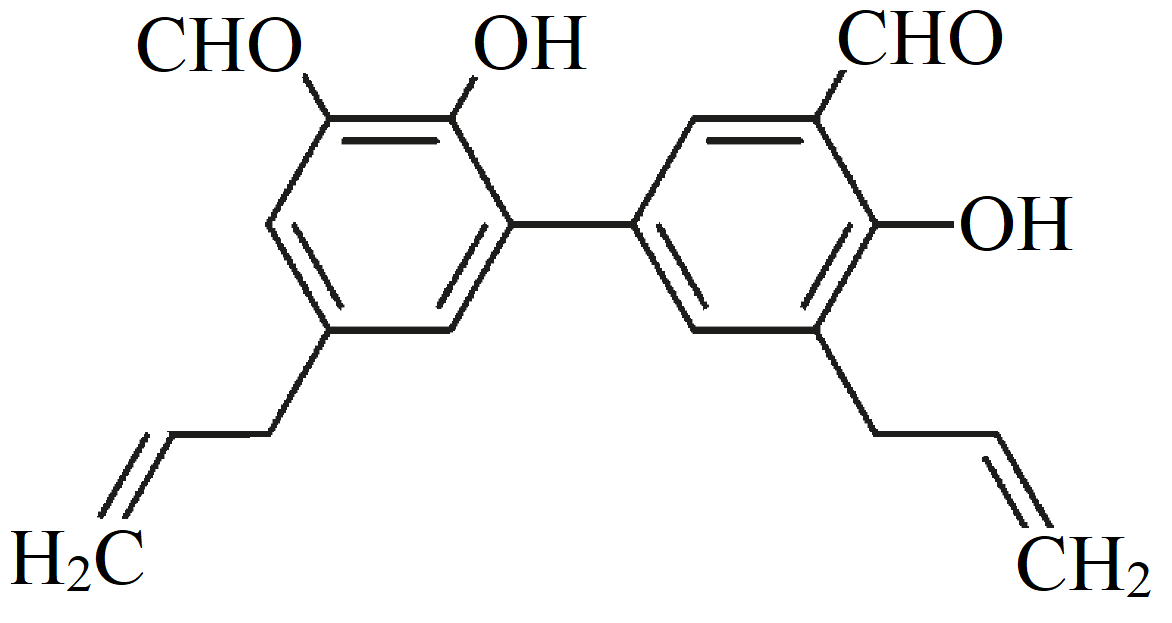


**ZDHXB-101**

**Fig. S1. Chemical structure of ZDHXB-101**

1. **Evaluating sEH inhibition**

**Methods**

The inhibition of soluble epoxide hydrolase (sEH) was determined using a sEH inhibitor screening assay kit (Cayman chem, MI, USA) according to the manual. AUDA is a sEH inhibitor, as a positive control drug. The fluorescence intensity was measured using a Gemini EM microplate reader (Meigu molecular instrument Co., LTD, USA) at an excitation and emission wavelengths of 320 nm and 465 nm, respectively. The formula is as follows:

Determine the average fluorescence (AF) of the background wells, 100% initial activity wells, and each of the inhibitors.

Subtract the background AF from the 100% initial activity and inhibitor AFs.

Use the following equation to calculate the percent activity remaining:


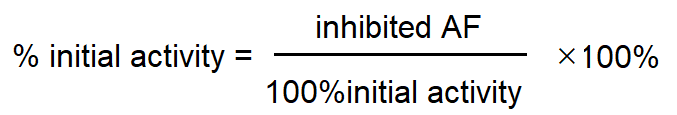


**Results:**

The results showed that the 50% inhibition (IC_50_) of initial activity of human recombinant sEH by ZDHXB-101 with 95% confidence limit was 1.23 (0.892~2.778) M, and AUDA was 1.38 (0.912~2.879) nM.

1. **Inhibiting cell proliferation and TGFβ1-induced sEH expression and increases 14 to 15-EET levels in human bronchial epithelial cell**

**Methods**

Cell culture: Human bronchial epithelial cell 16HBE was purchased from the Cell Bank, Chinese Academy of Sciences. The cells were cultured in 1640 (Hyclone) and F12 K medium(M&C GENE TECHNOLOGY(BEIJING) LTD., China) containing 10% fetal bovine serum (Hyclone), 2 mM [l-glutamine](https://www.sciencedirect.com/topics/pharmacology-toxicology-and-pharmaceutical-science/glutamine), 100 U/ml [penicillin](https://www.sciencedirect.com/topics/pharmacology-toxicology-and-pharmaceutical-science/penicillin-derivative) and 100 μg/ml [streptomycin](https://www.sciencedirect.com/topics/earth-and-planetary-sciences/streptomycin). Fresh media were replenished every 2 to 3 days. For each experiment, the cells (2 × 10^5^ cells/ml) were plated in 6-well culture plates. 16HBE cell was continuously maintained at 37 °C in a humidified atmosphere of 5% CO_2_. The cells were treated with pharmacological [inhibitors](https://www.sciencedirect.com/topics/earth-and-planetary-sciences/inhibitor), as follows: [TGF-β](https://www.sciencedirect.com/topics/pharmacology-toxicology-and-pharmaceutical-science/transforming-growth-factor-beta)_1_ (Sino Biological. Inc, China) 2.5, 5 and 10 ng/ml; sEH inhibitor ZDHXB-101 or AUDA (5, 10 and 20 μM) for 30 min. 16HBE cell was plated in 6-well plates at 2 × 10^5^ cells per well and rendered quiescent by incubation at 37 °C for 24 h. Then starved cells were exposed to ZDHXB-101 and AUDA (5, 10, 20 μM) and TGF-β (2.5, 5, 10 ng/ml) for 48 h prior to analysis.

Cell viability, proliferation, and EET level assay: The [viability](https://www.sciencedirect.com/topics/earth-and-planetary-sciences/viability) levels of 16HBE cell at the logarithmic phase were determined using the MTT assay. Briefly, cells (1.5 × 10^4^ cells/ml) were incubated for 24 h with different concentrations of ZDHXB-101 and AUDA (5, 10, and 20 μM). After various treatments, the medium was removed, and the cells were incubated with a solution of 1 mg/ml MTT (Sigma, St. Louis, MO, USA) for 4 h. Finally, the supernatant was removed, and [dimethyl sulfoxide](https://www.sciencedirect.com/topics/pharmacology-toxicology-and-pharmaceutical-science/dimethyl-sulfoxide) (DMSO, Sigma, St. Louis, MO, USA) was added to solubilize the [resultant](https://www.sciencedirect.com/topics/earth-and-planetary-sciences/resultant) [formazan](https://www.sciencedirect.com/topics/pharmacology-toxicology-and-pharmaceutical-science/formazan) [salt](https://www.sciencedirect.com/topics/pharmacology-toxicology-and-pharmaceutical-science/inorganic-salt). The amount of formazan salt was determined by measuring the absorbance at 490 nm using a [microplate](https://www.sciencedirect.com/topics/earth-and-planetary-sciences/microplate) reader (Bio-Rad 680, USA). The viability of the cells was quantified as a percentage compared to the blank (ZDHXB-101or AUDA cencentration at 0). The [lactate dehydrogenase](https://www.sciencedirect.com/topics/pharmacology-toxicology-and-pharmaceutical-science/lactate-dehydrogenase) (LDH) levels of 16HBE cell at logarithmic phase was determined using an ELISA kit (Jiancheng Bioengineering Institute, Nanjing, Jiangsu Province, China). 16HBE cell was plated in 6-well plates at 2 × 10^5^ cells per well and rendered quiescent by incubation at 37 °C for 24 h. Then starved cells were exposed to ZDHXB-101or AUDA (5, 10, 20 μM) and TGF-β (0, 1.25, 2.5, 5, 10 ng/ml) for 4 h prior to analysis. The protein levels of sEH were assessed by Western blot. The 14, 15-EETs levels of cell supernatant of were measured using a 14, 15-EET/DHET ELISA kit (Detroit R&D Inc., Detroit, MI, USA) according to the manufacturers’ manual.

**Results**

ZDHXB-101or AUDA 5, 10, and 20 μM concentrations suppressed changes in [cell proliferation](https://www.sciencedirect.com/topics/pharmacology-toxicology-and-pharmaceutical-science/cell-proliferation) in 16HBE cells ([Fig. S2](https://www.sciencedirect.com/science/article/pii/S0300483X17301828?via%3Dihub#fig0030)A and B), but not inhibited cell activity except AUDA at 20 μM concentration ([Fig. S2](https://www.sciencedirect.com/science/article/pii/S0300483X17301828?via%3Dihub#fig0030)C). TGF-β1 induced an increase in sEH protein expression (Fig.S2D), which was reversed by ZDHXB-101or AUDA 10 μM in 16HBE cells (Fig.S2E). TGF-β1 markedly reduced the EET levels (Fig.S2F). The ZDHXB-101or AUDA-treated cells not only had significantly increased EET levels, but also reversed effect of TGF-β1 compared with the vehicle group (Fig.S2F).


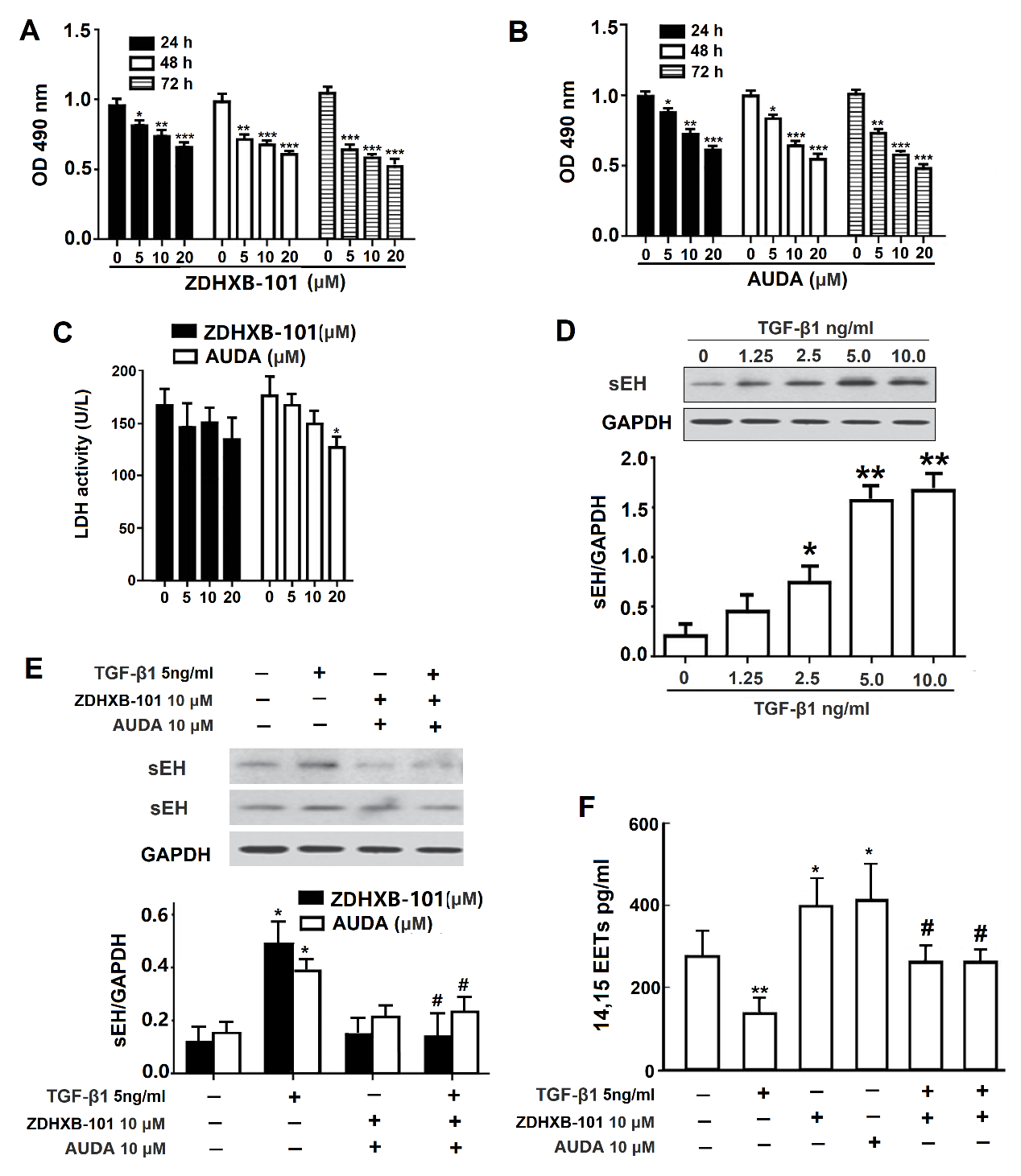


Fig. S2. Effects of ZDHXB-101 on [cell proliferation](https://www.sciencedirect.com/topics/pharmacology-toxicology-and-pharmaceutical-science/cell-proliferation) and activity in 16HBE cells, inhibits TGFβ1-induced increase in soluble [epoxide](https://www.sciencedirect.com/topics/earth-and-planetary-sciences/epoxy-compounds) hydrolase (sEH) expression and decrease in 14, 15-EETs levels. (A and D) [cell proliferation](https://www.sciencedirect.com/topics/pharmacology-toxicology-and-pharmaceutical-science/cell-proliferation); (C) cell activity; (D and E) sEH protein expression; (F) 14, 15-EETs level. 16HBE cell was treated with the indicated concentrations (5, 10, 20 μM) of ZDHXB-101or AUDA for 24–72 h. The [viability](https://www.sciencedirect.com/topics/earth-and-planetary-sciences/viability) levels of 16HBE cells at the logarithmic phase were determined using the MTT assay (n = 6 per group). The [lactate dehydrogenase](https://www.sciencedirect.com/topics/pharmacology-toxicology-and-pharmaceutical-science/lactate-dehydrogenase) (LDH) levels were determined using ELISA assay (n = 6 per group). (D and E) The sEH expression of 16HBE cells were induced with the indicated concentrations (1.25–10 μM) of TGFβ1 for 24 h. The protein levels of sEH were assessed by [western blot](https://www.sciencedirect.com/topics/pharmacology-toxicology-and-pharmaceutical-science/western-blot). The 14, 15-EETs levels were determined using ELISA assay (n = 6 per group). The data represent mean ± [S.E.M.](https://www.sciencedirect.com/topics/earth-and-planetary-sciences/scanning-electron-microscopy) from 4 independent experiments, ^*^p < 0.05, ^**^p < 0.01 and ^***^p < 0.001 compared with the untreated group. ^#^p < 0.05 indicates significant differences between the TGFβ1 group and the TGFβ1 + AUDA group.
